# Unhappy doctors? A longitudinal study of life and job satisfaction among Norwegian doctors 1994 – 2002

**DOI:** 10.1186/1472-6963-5-44

**Published:** 2005-06-08

**Authors:** Magne Nylenna, Pål Gulbrandsen, Reidun Førde, Olaf G Aasland

**Affiliations:** 1 Department of Public Health and General Practice, Norwegian University of Science and Technology, N-7489 Trondheim, Norway; 2 Directorate for Health and Social Affairs, PO Box 7000, St Olavs plass, N-0130 Oslo, Norway; 3 Medical Faculty Division, Akershus University Hospital, University of Oslo, Sykehusveien 27, N-1434 Nordbyhagen, Norway; 4 The Research Institute of the Norwegian Medical Association, PO Box 1152 Sentrum, N-0107 Oslo, Norway; 5 Section for Medical Ethics, Department of General Practice and Community Medicine, University of Oslo, P.O.Box 1130 Blindern, N-0318 Oslo, Norway; 6 Department of Health Management and Health Economics, University of Oslo, PO Box 1089, Blindern, N-0317 Oslo, Norway

## Abstract

**Background:**

General opinion is that doctors are increasingly dissatisfied with their job, but few longitudinal studies exist. This study has been conducted to investigate a possible decline in professional and personal satisfaction among doctors by the turn of the century.

**Methods:**

We have done a survey among a representative sample of 1 174 Norwegian doctors in 2002 (response rate 73 %) and compared the findings with answers to the same questions by (most of) the same doctors in 1994 and 2000. The main outcome measures were self reported levels of life satisfaction and job satisfaction according to the Job Satisfaction Scale (JSS).

**Results:**

Most Norwegian doctors are happy. They reported an average life satisfaction of 5.21 in 1994 and 5.32 in 2002 on a scale from 1 (extremely dissatisfied) to 7 (extremely satisfied). Half of the respondents reported a very high level of general life satisfaction (a score of 6 or 7) while only one third said they would have reported this high level of satisfaction five years ago. The doctors thought that they had a higher level of job satisfaction than other comparable professional groups. The job satisfaction scale among the same doctors showed a significant increase from 1994 to 2002. Anaesthesiologists and internists reported a lower and psychiatrists and primary care doctors reported a higher level of job satisfaction than the average.

**Conclusion:**

Norwegian doctors seem to have enjoyed an increasing level of life and job satisfaction rather than a decline over the last decade. This challenges the general impression of unhappy doctors as a general and worldwide phenomenon.

## Background

"Doctors are unhappy.... The unhappiness has been illustrated in a plethora of surveys...", Richard Smith, the then editor of *BMJ*, concluded a few years ago [1]. "Anecdotes and an expanding body of empirical data suggest a widespread professional malaise", Abigail Zuger wrote in *The New England Journal of Medicine *last year [2]. Doctor discontent is a current topic for discussion in medical journals, and there seems to be a general agreement – at least within the profession itself – that job satisfaction among physicians is declining. But how well founded on empirical investigations is this impression?

A literature search through PubMed [3] in January 2005 on the combination "discontent + physician" revealed 41 English language papers published during the last 25 years. Most of them were editorials and commentaries. Only five of the publications were based on original data on doctors' satisfaction [4-8]. The only evidence of a negative trend over time among the 41 publications was given by Murray et al. [8]. They found a declining satisfaction with most areas of practice among general internists and family practitioners in Massachusetts, USA, based on data from two cross-sectional studies in 1986 and 1997 respectively [8]. Even though there must be other reports than found by this search, there is reason to believe that the current debate is based more on opinion than on evidence.

In Norway, doctors' job satisfaction and subjective well-being have been studied since 1994 as part of an extensive research programme on physicians' health, sickness, and working conditions [9]. A representative reference panel has been surveyed on a regular basis [10]. The latest survey was undertaken in 2002 and includes Norwegian doctors' self-reported happiness and how they consider their own job satisfaction as compared with their belief about the job satisfaction of other professionals. A comparison with data from similar surveys in 1994 and 2000 might show whether there had been a shift over time.

## Methods

The Research Institute of The Norwegian Medical Association recruited in 1993 a Reference Panel by inviting a random sample of 2 000 active doctors to participate. 1 272 doctors agreed, and they have since received questionnaires more or less annually. 21 doctors have dropped out, due to death or voluntary withdrawal. In January 2000 another 795 randomly selected doctors who had received their license after 1993 were invited to join the panel, of which 365 agreed. Hence the number of panellists by primo 2000 was 1 616. Between 2000 and 2002 ten doctors died or withdrew from the panel. The remaining 1 606 Norwegian doctors were sent an 11-page questionnaire in June 2002 with one reminder in August, including several questions on subjective well-being:

- On a three point scale (less satisfied, as satisfied, more satisfied) they were asked to compare their own job satisfaction with the one they believe applies to nine other professional groups.

- They were asked to rate on a scale from 1 (extremely dissatisfied) to 7 (extremely satisfied) the question: "When you think about your life at the moment, would you say that by and large you are satisfied with life or are you mostly dissatisfied?" Many of the same doctors had also answered this question in 1994. On a similar scale they were asked to indicate how they would have answered this question five years ago and how they expected to rate it five years ahead. The distribution of responses to these questions is sufficiently close to normal to use parametric tests.

- Ten questions on different aspects of working conditions (responsibility, variation, collaborators, physical working conditions, possibility of using own skills, choice between work methods, positive feedback for good work, pay and work hours) make up the job satisfaction scale (JSS) based on the work by Warr et. al. [11]. These questions are scored from 1 (extremely dissatisfied) to 7 (extremely satisfied) and add up to a scale with a theoretical range from 10 (dissatisfied with job) to 70 (satisfied with job). The distribution is close to normal. JSS results were also available for 1994 and 2000 from most of the respondents.

The 43 medical specialties were reduced to nine categories: no speciality, specialists in family medicine, laboratory medicine (including pathology and radiology), internal medicine (including paediatrics and neurology), surgery, anaesthesiology, gynaecology and obstetrics, psychiatry and public health. Comparisons of the scales over time were made with GLM (general linear modelling) repeated measures, with age as covariate (to control for possible age effects) and between groups with error bar plots. Differences in categorical variables were given as proportions (percentages) with 95% confidence intervals. The analyses were performed with SPSS 12.

## Results

A total of 1 174 doctors completed the questionnaire (369 women (31 %), 805 men (69 %)) which gave a response rate of 73 %. For 874 of the doctors we also had life satisfaction data from 1994. For 886 of the doctors we had job satisfaction data from 1994 and 2000, for 981 we had such data from 2000 and 2002, and for 841 from 1994 and 2002. 722 doctors were registered at all three points in time.

With the exception of aviation pilots, who were believed to be on the same level of satisfaction, the respondents thought themselves to be more satisfied with their job than six other professional groups mentioned (lawyers, clergymen, psychologists, craftsmen, farmers, policemen/-women). Three out of four and two out of three, respectively, believed themselves to be more satisfied than teachers and nurses. 67 % would have chosen a medical career over again if they were young today.

The reported level of subjective well-being was high, with 51.3 % (95 % CI 48.4 to 54.3) of the doctors reporting very high life satisfaction (6 or 7 on a scale from 1 to 7). When asked what they would have answered five years ago only 35.3 % (32.5 to 38.1) reported the same high level of satisfaction. The expected life satisfaction five years ahead did not differ from the present situation (51.5% expected a very high life satisfaction (48.4 – 54.5)). The doctors (N = 873) who had answered the question on subjective well-being eight years earlier had an estimated average score of 5.26 (5.19 to 5.33) in1994 and 5.34 (5.26 to 5.42) in 2002.

**Figure 1:**
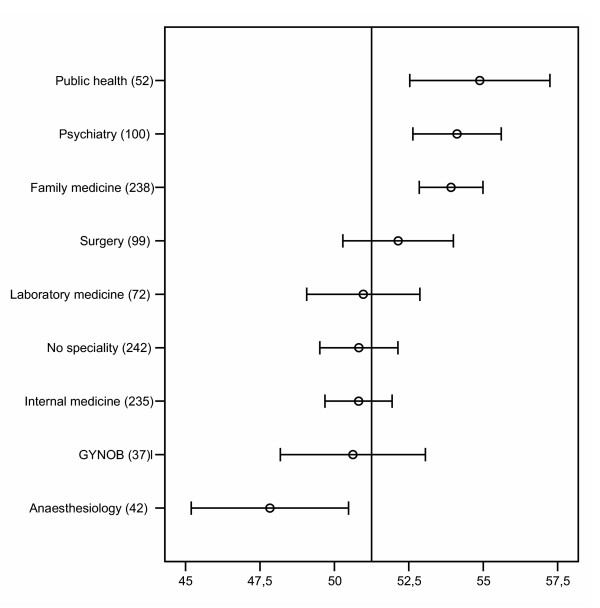
Job satisfaction scale among 1 117 Norwegian doctors in different specialty groups in 2002 (numbers (N) in parentheses). Means and 95% confidence intervals. The vertical line indicates the overall mean. Higher values mean higher level of satisfaction.

The 2002 survey showed an average of 52.0 on the job satisfaction scale (range 10 to 70). There was a moderate positive correlation with age (job satisfaction increased with increasing age) (r = .17, p < .0001) but no significant gender difference. Means of the different specialist groups are shown in figure 1. Anaesthesiologists and specialists in internal medicine scored significantly lower than the overall mean, while psychiatrists and specialists in family medicine and public health scored significantly higher.

A GLM analysis of the doctors who answered the same questions at all three points in time showed an estimated mean difference between 1994 and 2000 of 1.37 (.71 to 2.03), and between 1994 and 2002 of 1.09 (.42 to 1.76), i.e. positive and statistically significant changes.

**Figure 2:**
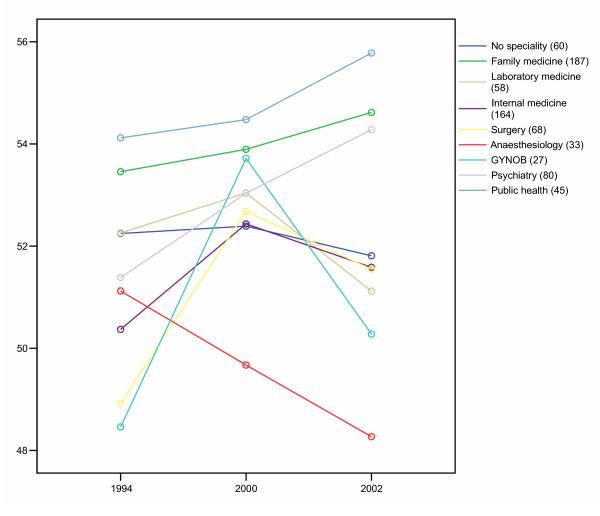
Job satisfaction scale among 722 Norwegian doctors in different specialty groups in 1994, 2000 and 2002. GLM-estimated means, controlled for age. Higher values mean higher level of satisfaction.

Figure 2 shows changes in estimated JSS-means over time for the same 722 doctors in nine different specialty groups. Anaesthesiologists show a steady decrease, whereas psychiatrists and specialists in family medicine and public health show a steady increase in job satisfaction.

## Discussion

Our findings do not confirm a declining degree of happiness among Norwegian doctors over the last eight years. Data from 2002 show a high level of general satisfaction. Over half of the doctors regarded themselves as very satisfied with life and they believed that they are more satisfied with life now than five years ago. Answers to the identical question on present life satisfaction among the same doctors show no decline over an eight-year period. Most of the doctors believed that they had a higher level of job satisfaction than other professional groups. A comparison of reported job satisfaction among the same doctors between 1994 and 2002 showed an increasing rather than a declining level over the years, but there were interesting differences among specialties. Anaesthesiologists and other hospital-based doctors did not show the trend of increasing job satisfaction seen among psychiatrists and doctors who work outside hospitals. Our next surveys may show whether this difference is increasing.

Although this is one of the few longitudinal studies on doctor satisfaction it has several limitations, the most important being the validity and reliability of questionnaire data on subjective well-being. Reliability tests suggest that some respondents indicate quite different levels of satisfaction over a very short period of time. On the other hand, our samples and scales should be large enough to account for this, larger than those of Murray et al. [8], and as opposed to them we are actually following the same doctors over time. Another limitation lies of course in the response rate and to what extent our respondents are representative of doctors in general. However, our response rates are higher than in most comparable studies, and we have no reason to believe that our panel is not representative of Norwegian doctors, as has been discussed elsewhere [12].

In spite of the impression created by the medical community most physicians continue to report overall career satisfaction [13]. A recent study of doctors who graduated from UK medical schools in 1974 showed a high level of job satisfaction, with a median score of 18 – 19 on a scale from 5 to 25 [14]. Even in the USA, where discontent has become an accepted characterization of doctors' attitude [2,15], satisfaction levels have not changed dramatically [16], though there are reports of a declining career satisfaction related to managed care and decreasing professional autonomy [17,18].

The *BMJ *has, through a widely cited editorial [1] and a highly selective and non-scientific Internet survey [19], more or less established as an indisputable fact that "unhappy doctors are a worldwide phenomenon" [20]. Our findings challenge the evidence for this statement. No doubt many doctors are discontented with present developments in health care, but the picture is more complex, and at an individual level most Norwegian doctors are still satisfied both with their private and professional life. This accords well with a general paradox: most attention in the press and by the public is given to misery and displeasure, though conversely people across the world report a high degree of happiness whenever they are questioned directly [21].

## Conclusion

Most doctors are happy. No decline in professional and personal satisfaction could be found over the last decade. Norwegian doctors had a higher level of job satisfaction in 2002 than in 1994. There were important differences among specialties; general practitioners and psychiatrists were significantly more happy than other doctors. Anaesthesiologists and other hospital-based doctors did not show the trend of increasing job satisfaction found among psychiatrists and primary care doctors.

## Competing interests

The author(s) declare that they have no competing interests.

## Authors' contributions

MN, PG, RF and OGAa jointly conceived the idea for the study. OGAa analysed the data and all authors interpreted the results. MN drafted the manuscript. All authors revised the paper and approved the final version. MN and OGAa are guarantors.

## Pre-publication history

The pre-publication history for this paper can be accessed here:


